# *Pru p 3* oral immunotherapy efficacy, induced immunological changes and quality of life improvement in patients with LTP syndrome

**DOI:** 10.1186/s13601-020-00325-y

**Published:** 2020-06-08

**Authors:** Alejandra González Pérez, Antonio Carbonell Martínez, Ana Isabel Escudero Pastor, Cristina Navarro Garrido, Juan Carlos Miralles López

**Affiliations:** Allergy Section, Reina Sofía University General Hospital, Intendente Jorge Palacios Avenue, 1, 30003 Murcia, Spain

**Keywords:** Peach allergy, *Pru p 3* allergy, *Pru p 3* oral immunotherapy, LTP syndrome, Lipid transfer proteins

To the editors,

Lipid transfer protein (LTP) syndrome is the clinical situation present in patients with multiple sensitizations against LTP of different plant foods, due to the cross-reactivity between these components. A significant number of anaphylactic reactions have been described in people affected, aggravated or triggered on many occasions by the presence of cofactors [1, 2].

It is known that LTPs are panallergens that are responsible for severe allergic reactions to food, especially in Mediterranean countries where prevalence of the syndrome is much higher than in central and northern European countries.

According to the European Academy of Allergy position paper [3], food immunotherapy is indicated when avoidance measures are ineffective, undesirable, or cause serious limitations on patients’ quality of life.

A sublingually administered extract of the allergen *Pru p 3* (SLIT-peach, ALK-Abelló SA) has proven to be an effective treatment not only against peach allergy, but also against allergies to nuts such as peanuts [4], inducing a clearly positive regulatory T response with significant reduction of the TH2/TH9 response and markers of dendritic cell activation and maturation [5].

A specific unit for treating food allergies due to LTP sensitization has been created in the Allergy Department of the Reina Sofia Hospital in Murcia. Thus, in a study conducted under conditions of usual clinical practice, a 3 year treatment with SLIT-peach^®^ administered to patients with food allergy due to sensitization to *Pru p 3* have been evaluated.

SLIT was only prescribed to patients with anaphylaxis and/or urticaria-angioedema due to LTP sensitization.

Data from the first 18 patients who have completed 3 years of SLIT treatment were analyzed. The mean age was 31.4 ± 12.4 years (age range: 16–59 years) and 66.7% were women. We describe in Table 1 the type of pre-treatment reaction caused by food and the mean diameter of skin prick test to the involved food.

**Table 1:** Clinical manifestations before *Pru p 3* immunotherapy and results of skin tests with the foods involved

Food	Patients with anaphylaxis	Patients with urticaria/angioedema	Prick mean diameter (mm)
Peach	4	12	5.8
Apple	1	7	6
Kiwi	–	5	6.6
Banana	–	4	9
Grape	1	–	5
Strawberry	1	4	6.2
Melon	–	3	6.6
Nectarine	–	2	9
Pear	–	2	4.5
Cherry	1	–	4
Tomato	–	2	5.5
Onion	2	–	7
Lettuce	1	5	6
Almond	4	6	4.9
Sunflower seed	2	4	7
Peanut	2	11	6.6
Walnut	–	10	6.3
Hazelnut	–	8	8
Corn	2	–	13
Rice	2	1	11.3
Wheat	–	2	4
Bean	1	2	4.3
Pea	–	1	5

The presence of cofactors was detected In 10 patients (exercise in 4 cases, NSAIDs in 5 cases, and both cofactors in 1 case). In addition, 11 patients presented respiratory symptoms due to pollens (rhinitis in 7 cases and asthma in 4 cases).

A commercial extract of Prup3 (ALK-abello) with a concentration of 30 μg/ml was used for skin prick test (SPT). All patients had a positive LTP skin test and the mean diameter of the positive tests was 8 mm.

17 patients were sensitized to pollens: 10 patients to olive pollen, 9 to Artemisia vulgaris, 6 to Platanus, 4 to Parietaria and 2 to Cupressus. All of them contain LTP in their molecular structure.

Additionally, skin prick tests with natural foods were performed in all patients. The mean diameters of the positive skin tests to food are shown in Table 1.

An ISAC microarray was performed in the 18 patients included in our study: All of them were sensitized to *Pru p 3,* 13 patients were sensitized to *Ara h 9*, 11 patients to *Jug r 3*, 11 patient to *Cor a 8*, 10 patients to *Art v 3*, 8 patients to *Pla a 3*, 5 patients to *Ole e 7*, and 3 patients to *Tri a 14*.

Two patients were sensitized to kiwi thaumatin (non-LTP specific allergens).

The build-up phase was performed using a rapid 2-day schedule. Two patients had oral pruritus that required symptomatic treatment, so they were changed to a 4-day initiation schedule. There were no reactions during maintenance.

After 1 year of SLIT treatment, a single blind oral food challenge with peach was performed with negative results in all patients. Then, we progressively introduced the food involved in the previous reaction uneventfully. Only two patients who were sensitized to kiwi (thaumatin) had to keep avoiding this food.

The SLIT treatment was maintained for 3 years without interruption. After these 3 years of treatment, 16 patients kept eating all the foods that they could not previously tolerate. The other 2 patients tolerated all food but kiwi.

The mean score of the patients’ Food Allergy Quality of Life. Questionnaire-Adult Form (FAQLQ-AF) decreased from 140.6 to 83.2 after 3 years of treatment (p < 0.0001, Wilcoxon test for paired data). Furthermore, as shown in Table 2, significant changes in *Pru p 3* specific IgE and IgG4 levels were observed after 3 years of immunotherapy with peach extract.

**Table 2:** Statistical analysis of changes in *Pru p 3* specific IgE and IgG4 levels after *Pru p 3* immunotherapy

	Median (Q1, Q3)	p-value
IgE^a^ 0	6.51 (1.47, 21.70)	
IgE 1	3.85 (1.08, 10.70)	
IgE 3	4.69 (1.43, 8.76)	
Dif IgE year 1-basal	− 1.12 (− 11.00, 0.17)	0.1269
Dif IgE year 3-basal	− 1.85 (− 6.90, 0.30)	0.0448
IgG4^b^ 0	0.25 (0.10, 0.34)	
IgG4 1	1.01 (0.29, 3.37)	
IgG4 3	0.70 (0.24, 1.75)	
Dif IgG4 year 1-basal	0.49 (0.04, 3.27)	0.0007
Dif IgG4 year 3-basal	0.43 (0.12, 1.30)	0.0004

^a^ IgE: KU/l

^b^ IgG: mg/l

Every year, food allergies are the cause of increasing numbers of visits to allergy departments, with LTP syndrome being a significant factor behind this increase. The creation of specific food allergy units will lead to better care for these patients, who experience a significant impact on their quality of life as a result of their condition.

In this study conducted under the usual best clinical practice principles, it was found that treatment with SLIT-peach is effective from the first year of treatment, resulting in negative provocation. This effect is maintained after 3 years of treatment, with almost 90% of patients no longer needing any type of dietary restrictions and able to tolerate all of the foods they were unable to consume before. This fact has significantly contributed to improving the quality of life of the treated patients.

In vitro results are similar to those shown in previous studies, with a significant decrease in IgE and an increase in IgG4 specific to *Pru p 3* [4, 5].

In conclusion, this study conducted under the usual best clinical practice principles demonstrated tolerance induced by treatment with a sublingual *Pru p 3* extract to foods that had previously caused symptoms in patients with LTP syndrome, thus significantly improving their quality of life. Further studies will of course be necessary to verify the long-term effect of the treatment and the reintroduction of food.

## Data Availability

Contact A. González for an anonymized database.

## Abbreviations

LTP: Lipid transfer protein
OAS: Oral allergy syndrome
NSAIDs: Nonsteroidal anti-inflammatory drugs
FAQLQ-AF: Food Allergy Quality of Life. Questionnaire-Adult Form
